# Gold Nanoparticle-Coated ZrO_2_-Nanofiber Surface as a SERS-Active Substrate for Trace Detection of Pesticide Residue

**DOI:** 10.3390/nano8060402

**Published:** 2018-06-03

**Authors:** Han Lee, Jiunn-Der Liao, Kundan Sivashanmugan, Bernard Haochih Liu, Wei-en Fu, Chih-Chien Chen, Guo Dung Chen, Yung-Der Juang

**Affiliations:** 1 Department of Materials Science and Engineering, National Cheng Kung University, 1 University Road, Tainan 701, Taiwan; rick594007@hotmail.com (H.L.); sivashanmugannst87@gmail.com (K.S.); hcliu@mail.ncku.edu.tw (B.H.L.); mvp820820@gmail.com (C.-C.C.); 2 Medical Device Innovation Center, National Cheng Kung University, 1 University Road, Tainan 701, Taiwan; 3 Center for Measurement Standards, Industrial Technology Research Institute, No. 321, Kuang Fu Road, Sec. 2, Hsinchu 300, Taiwan; WeienFu@itri.org.tw (W.-e.F.); Eric_chen@itri.org.tw (G.D.C.); 4 Department of Materials Science, National University of Tainan, Tainan 700, Taiwan; juang@mail.nutn.edu.tw

**Keywords:** surface-enhanced Raman scattering, pesticide residue, gold nanoparticles, Zirconia nanofibers

## Abstract

Trace detection of common pesticide residue is necessary to assure safety of fruit and vegetables, given that the potential health risk to consumers is attributed to the contamination of the sources. A simple, rapid and effective means of finding the residue is however required for household purposes. In recent years, the technique in association with surface-enhanced Raman scattering (SERS) has been well developed in particular for trace detection of target molecules. Herein, gold nanoparticles (Au NPs) were integrated with sol-gel spin-coated Zirconia nanofibers (ZrO_2_ NFs) as a chemically stable substrate and used for SERS application. The morphologies of Au NPs/ZrO_2_ NFs were adjusted by the precursor concentrations (_X, X = 0.05–0.5 M) and the effect of SERS on Au NPs/ZrO_2_ NFs_X was evaluated by different Raman laser wavelengths using rhodamine 6G as the probe molecule at low concentrations. The target pesticides, phosmet (P1), carbaryl (C1), permethrin (P2) and cypermethrin (C2) were thereafter tested and analyzed. Au NPs/ZrO_2_ NFs_0.3 exhibited an enhancement factor of 2.1 × 10^7^, which could detect P1, C1, P2 and C2 at the concentrations down to 10^−8^, 10^−7^, 10^−7^ and 10^−6^ M, respectively. High selectivity to the organophosphates was also found. As the pesticides were dip-coated on an apple and then measured on the diluted juice containing sliced apple peels, the characteristic peaks of each pesticide could be clearly identified. It is thus promising to use NPs/ZrO_2_ NFs_0.3 as a novel SERS-active substrate for trace detection of pesticide residue upon, for example, fruits or vegetables.

## 1. Introduction

Agricultural production and safety, food industries, as well as the consumers are of great concern to governments [1]. The use of pesticides in agricultural products aims to increase the yield, as well as improve the quality of crops. Pesticide residue in food and in the environment provokes great public concern since it could pose potential health risks to not only the consumers but also the earth. Various analytical methods, including gas chromatography [2] and high-performance liquid chromatography [3], have been applied for trace detection of pesticides [4,5]. However, these methods are time consuming, labor intensive, and usually require complicated procedures for sample preparation. Thus, research has been conducted to develop advanced detection techniques instead of traditional methods to provide rapid, nondestructive food quality and safety evaluation and analysis for the industry [1].

As an emerging technology, surface-enhanced Raman scattering (SERS) techniques are becoming increasingly widespread and accessible for accurate and specific identification of chemical or microbiological contaminants in foodstuffs [6]. Over the past decades, the technique of SERS has been well developed as a sensitive and selective method, using, for example, nanostructured surfaces [7]. When target species with Raman-active modes are adsorbed on a nanostructured surface of noble metal(s), particular peak enhancement can be found in the Raman spectrum. Target species could be identified by verifying the specific fingerprints, which comprise information about vibrational modes in compounds. Advances in nanofabrication techniques have assisted the as-prepared substrates capable of lowering the detection limit of sensing target molecules or biomolecules. Particular enhancement of Raman-active modes is mainly attributed to two mechanisms: a chemical [8] and an electromagnetic (EM) effect [9]. For the former, as the target molecules are chemically adsorbed upon a SERS-active substrate, Raman-active modes in molecules can be enhanced in different degrees, depending on the distances of the modes with respect to the substrate surface [10]. For the latter, a strong EM may be generated from the morphologies of nanostructures. As the target species is trapped/adhered to the proximity of a metal nanostructure (usually Au, Ag or Cu), Raman intensity is enhanced due to the amplification of the EM field resonance, not only by the size and shape of the nanostructure, but also the interactions among Raman laser wavelength, the target species, and the substrate surface in the microenvironment [11].

Researchers have investigated the formation of hot spots, which are small regions of a highly enhanced EM field that leads to high enhancement factor (EF). These regions can be observed or even predicted using computer simulation and modeling to describe the quantum effect on subnanometer gaps [12]. Usually, a noble metal surface with the structure of high roughness [7], edges [13], hollow cavities [14], and so on, is advantageous to produce EM as well as surface plasmon [15]. Strategies for fabricating nanostructures can be categorized as, for example, top-down [4], bottom-up [16], combination [17], and template-assisted techniques [18]. In addition, for example, Zirconia (ZrO_2_), especially the thin-film type, is one of the promising common materials used for a base SERS-active substrate. [19] By manipulating the structure of ZrO_2_ into, for example, a nanofiber, a large surface area is gained. As the ZrO_2_ nanofibers’ (ZrO_2_/NFs’) structure is combined with gold nanoparticles (Au NPs) to gain the EM effect between NPs or intra-Au NPs [13], the hybrid nanostructure may increase EM at the metal–semiconductor junction and generate a strong local EM field at the interface. [20] In addition, Au NPs embedded in ZrO_2_ NFs are competent to prevent the electron-hole pairs from recombination; thus a significant enhancement of SERS is anticipated.

In this work, ZrO_2_ NFs are therefore made by a spin-coated sol-gel method and formed as templates; subsequently, Au NPs are deposited upon ZrO_2_ NFs. The as-designed Au NPs embedded upon ZrO_2_ NFs as the substrate (denoted as Au NPs/ZrO_2_ NFs) is proposed. The structure of NFs is designed to increase the surface areas for detecting the target species, while the embedded Au NPs upon the as-formed ZrO_2_ NFs are anticipated to gain the additional effect of SERS. Thereafter, different pesticides at low concentrations are tested and measured to interpret their competences for trace detection of pesticide residue.

## 2. Experimental Section

### 2.1. Fabrication of ZrO_2_ NFs and NPs/ZrO_2_ NFs

**Figure 1:**
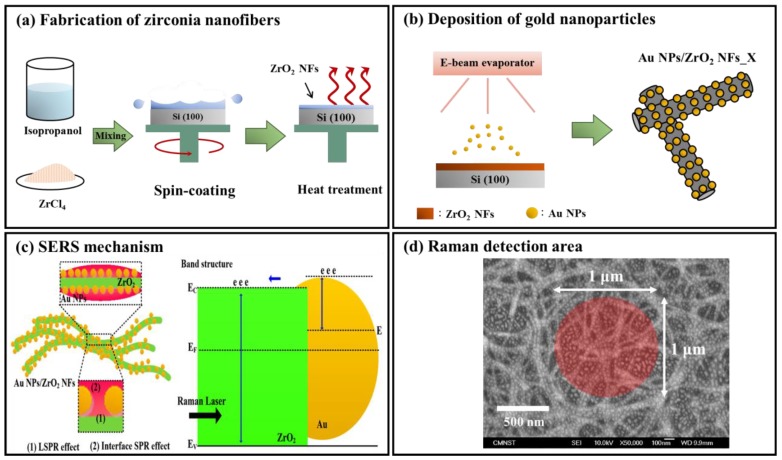
(**a**) Steps for preparing Au NPs/ZrO_2_ NFs: 1. mixing isopropanol with ZrCl_4_; 2. forming ZrO_2_ thin film by spin-coating method; 3. forming ZrO_2_ NFs by removing solvents; (**b**) steps for depositing Au NPs upon ZrO_2_ NFs by e-beam evaporator; (**c**) SERS mechanism based on Au NPs deposited upon random ZrO_2_ NFs; (**d**) SERS signals from Au NPs deposited upon ZrO_2_ NFs, no Raman signal from the surface of Au NPs upon Si (100) and ZrO_2_ NFs without the integration of Au NPs.

Zirconium tetrachloride (ZrCl_4_, 98%, Acros Organics, Geel, Belgium) was used as the precursor for the synthesis of ZrO_2_. The precursor solutions with various concentrations were prepared by dissolving appropriate amounts of ZrCl_4_ in 10 mL isopropanol (99.8%, Panreac AppliChem Barcelona, Spain). After vigorous stirring, the precursor solutions were stored in sealed glassware and aged at room temperature for 24 h. 100 μL of the precursor solution was spin-coated onto a 2 cm × 2 cm silicon wafer at 2500 rpm for 30 s at an ambient temperature and relative humidity of 25 °C and 70%, respectively. Prior to coating, silicon wafers were pre-cleaned with hydrochloric acid (37%, Panreac AppliChem, Barcelona, Spain) and then ethanol (99.9%, Merck KGaA, Darmstadt, Germany) to remove the organic contaminants on the surface. The as-prepared samples were heated at 100 °C for 10 min to evaporate the solvent and then calcined at 500 °C for 3 h in the air for densification. The as-formed ZrO_2_ NFs are distinguished as ZrO_2_ NFs_X, where X is the concentration of precursor in M. The fabrication procedures of ZrO_2_ NFs are simply illustrated in Figure 1a.

Au NPs were then deposited onto ZrO_2_ NFs by using an electron beam evaporator (VT1-10CE, ULVAC Inc., Chigasaki, Japan) with a thickness of about 1.5 nm and at a rate of 0.1 Å/s. The deposition was operated under ultra-high vacuum conditions maintained below 7 × 10^−6^ torr. The SEM images after deposition are shown in Supporting Data 1 (a) with a magnification of 5 × 10^5^, and Supporting Data 1 (b) with a higher magnification of 10^6^. The results show that the nano-sized Au NP gold particles closely arranged on the nanofiber surface. In addition, the Energy Dispersive Spectroscopy (EDS) mapping image of the cross-sectioned sample AuNPs/ZrO_2_NFs_0.3 is shown in the Supporting Data 2. The particle-size distribution of the nano-Au NPs with the range from 30 to 45 nm is also shown in the Supporting Data 3, in which a narrow distribution curve represents a uniform size of Au NPs. The as-formed samples were distinguished as Au NPs/ZrO_2_ NFs_X, where X is the concentration of precursor in M. The fabrication of Au NPs/ZrO_2_ NFs_X is simply illustrated in Figure 1b. As illustrated in Figure 1c with Au NPs deposited upon ZrO_2_ NFs, intra-Au NP interactions (marked “1” in the figure) tend to be much stronger than electron transfer at the junction. In case of Au NPs distributed over ZrO_2_ NFs (marked “2” in the figure), there exists a recombination of electron-hole pairs, leading to a significant improvement of SERS properties. As appropriate laser wavelength is applied, surface plasmon resonance of Au NPs and hot spots between Au NPs may presumably occur and contribute to the effect of SERS.

### 2.2. Structural and Morphological Characterization

The compositions of ZrO_2_ NFs and NPs/ZrO_2_ NFs were analyzed by an X-ray diffractometer (XRD, MiniFlex II, Rigaku, Japan) using CuKα radiation with scanning angles ranging from 20° to 61.5°. The obtained XRD patterns were compared with JCPDS card No. 89-6976 [21] and 65-1022 [22]. The photo-images of the surfaces of ZrO_2_ NFs and NPs/ZrO_2_ NFs were taken by a high-resolution thermal field emission scanning electron microscope (FE-SEM, JSM-7000, JEOL, Tokyo, Japan), which was operated at an accelerating voltage of 10 kV. All the samples subsequent to FE-SEM were platinum-coated in advance. The dimensions of ZrO_2_ NFs and Au NPs were thereafter presented and determined by FE-SEM photo-images and software Image J (National Institutes of Health, USA).

### 2.3. Enhancement Evaluation for the Effect of SERS

The test probe molecule, rhodamine 6G (R6G), was used as the referenced target species. R6G was diluted in aqueous solution to a concentration of 10^−3^ M as the standard solution. A quantity of 5 μL R6G standard solution was then placed on each substrate and dried at room temperature for subsequent analysis. A Raman spectrum was obtained by using Raman spectrometer with a confocal microscope (Renishaw, United Kingdom). He-Ne and diode lasers with excitation wavelengths of 633 and 785 nm were respectively applied. An air-cooled CCD was used as the detector and the incident power was about 3 mW. The samples were scanned with an exposure time of 10 s over an area of 1 μm × 1 μm (the size of the laser spot was 1 μm, as shown in Figure 1d), using a 50× objective. The SERS spectra were averaged from 10 consecutive measurements on different samples. All Raman spectra were normalized by using the peak fit software.

### 2.4. Trace Detection of Pesticide Residue and Those on Apples

The optimal SERS-active substrates were thereafter examined using four types of pesticides, namely phosmet (P1), carbaryl (C1), permethrin (P2) and cypermethrin (C2). An aqueous solution of each pesticide was diluted to concentrations ranging from 10^−2^ to 10^−10^ M. To verify the effect of SERS with respect to organophosphates, mixed pesticides were prepared to the concentration of 10^−3^ M. A quantity of 5 μL from single and diluted pesticide and mixed pesticides were respectively placed on the substrates and dried at room temperature for subsequent analyses. The test apples were purchased from a local market and then immersed in a solution of each pesticide in 10^−2^ M. The apple containing pesticide on the surface was thereafter cut into pieces. Each pesticide on the outer surface of the sliced apple was extracted by soaking a 1 cm × 1 cm apple peel in 500 μL ethanol. The as-formed mixture was then vigorously shaken for 10 min and subjected to 4500 rpm of centrifugation for 5 min. A final product with a quantity of 5 μL was placed on the respective substrates and dried at room temperature for subsequent analyses.

## 3. Results and Discussion

### 3.1. The Quality of ZrO_2_ NFs and Au NPs/ZrO_2_ NFs

**Figure 2:**
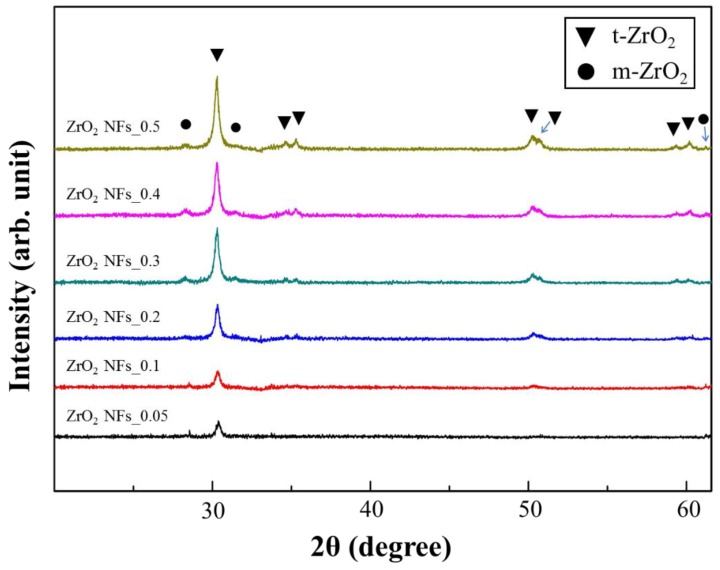
XRD patterns of ZrO_2_ NFs with different ZrCl_4_ concentrations.

In Figure 2, XRD patterns of ZrO_2_ NFs obtained from the various precursor concentrations are shown. Characteristic peaks of tetragonal zirconia, namely (011), (002), (110), (112), (020), (013) and (121) resulted. Additional peaks from monoclinic zirconia, including (−111), (111) and (311), were also observed. The result indicates that ZrO_2_ is successfully prepared by a spin-coated sol-gel method. Nevertheless, the intensity of the characteristic peaks is lowered with the decreased concentration of precursor solution as the coverage of the generated ZrO_2_ is still insufficient.

**Figure 3:**
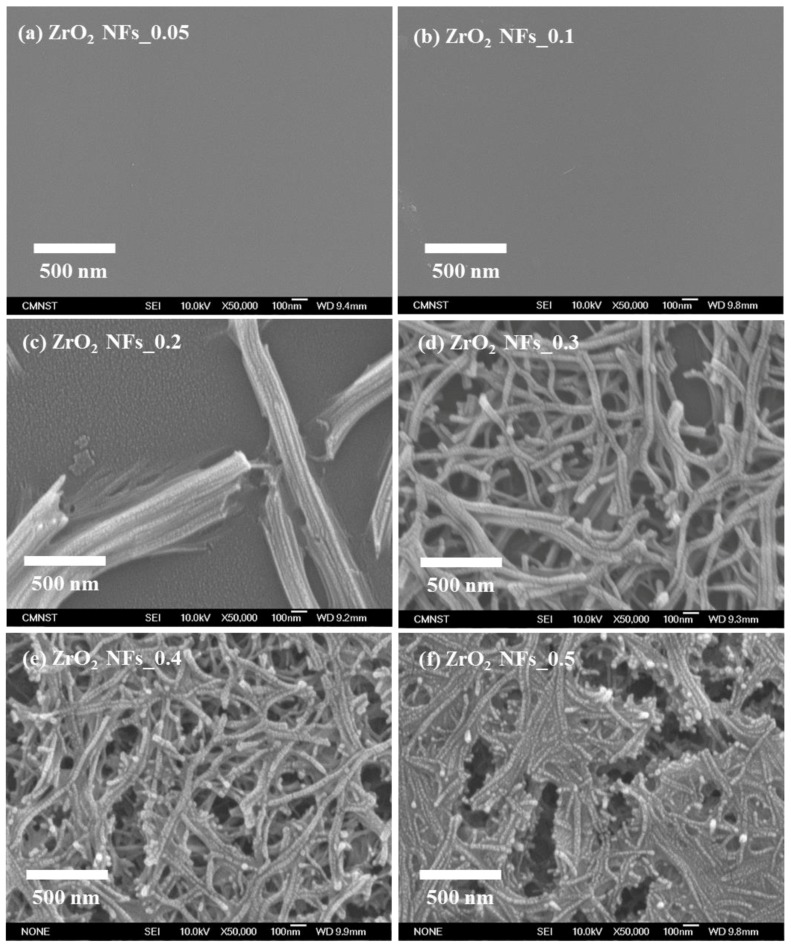
SEM micrographs of ZrO_2_ NFs with different ZrCl_4_ concentrations. Morphologies from the surfaces of NPs/ZrO_2_ NFs_X with (**a**) X = 0.05 (**b**) X = 0.1, (**c**) X = 0.2, (**d**) X = 0.3, (**e**) X = 0.4, and (**f**) X = 0.5.

In Figure 3, FE–SEM photo-images of Au NPs/ZrO_2_ NFs were obtained from various precursor concentrations, corresponding to their XRD patterns (ZrO_2_ NFs) shown in Figure 2. At the beginning, the base ZrO_2_ NFs were significantly generated, as the precursor concentrations were higher than 0.2 M (i.e., ZrO_2_ NFs_0.2, _0.3, _0.4, and _0.5); the diameter of ZrO_2_ NFs was measured around 35 to 50 nm. With the decreased precursor concentrations, that is, ZrO_2_ NFs_0.05 and _0.1, the surface of ZrO_2_ NFs was broad and flat, as shown in Figure 3a,b. From Figure 3c–f, as the precursor concentration was increased, the hydrolytic rate was decreased; the morphology of the base ZrO_2_ NFs became agglomerated and connected with thinner nanofibers. As the increase of surface areas for the subsequent Au NP deposition is of significance, ZrO_2_ NFs with distinct size and dimension of nanofibers are preferable in this study. Thereafter, the sample Au NPs/ZrO_2_ NFs_0.3 (Figure 3d) suitably resulted. As determined by software Image J from 200 random Au NPs, the diameter of Au NPs in the range from 30 to 45 nm was averaged, which was corresponding to the rough diameter of ZrO_2_ NFs.

### 3.2. The Effect of SERS for the Samples of Au NPs/ZrO_2_ NFs

**Figure 4:**
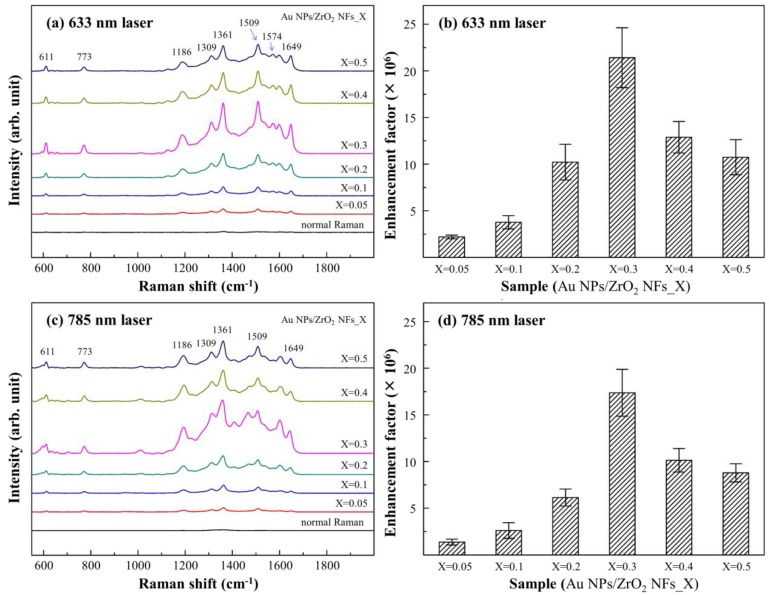
The effect of SERS on Au NPs deposited upon random ZrO_2_ NFs with different ZrCl_4_ concentrations were evaluated using the molecular probe R6G and different Raman laser wavelengths. (**a**) and (**b**) are the intensity and enhancement factor of 633 nm laser; similarly to (**c**) and (**d**) with 785 nm laser.

In Figure 4, SERS spectra were taken from the samples of Au NPs/ZrO_2_ NFs with the test molecule R6G of 10^−3^ M attached to them. The Raman laser with the wavelengths of 633 (Figure 4a) and 785 nm (Figure 4c) were respectively used. The characteristic peaks of R6G upon Au NPs/ZrO_2_ NFs were significantly detected and enhanced, as compared to the flat substrate. The most intense peak at 1361 cm^−1^, which is assigned to the stretching of C–C in aromatics, is usually used to calculate the EF. By taking the peak at 1361 cm^−1^ as the reference, the peak enhancement by 633 nm laser wavelength was higher than that by 785 nm. In Figure 4b,d, Au NPs/ZrO_2_ NFs_0.3 showed the most intense EF of 2.1 × 10^7^ under the excitation of 633 nm laser wavelength, which is corresponding to the sample with distinct size and dimension of nanofibers, shown in Figure 3d. Particularly, the gaps between the aggregated Au NPs generate abundant “hot-spot” structures for SERS, which are homogeneously distributed on the substrate, making a robust and reproducible enhancement of the Raman signal feasible.

### 3.3. Trace Detection of Pesticides Using the Optimal Au NPs/ZrO_2_ NFs_0.3

**Figure 5:**
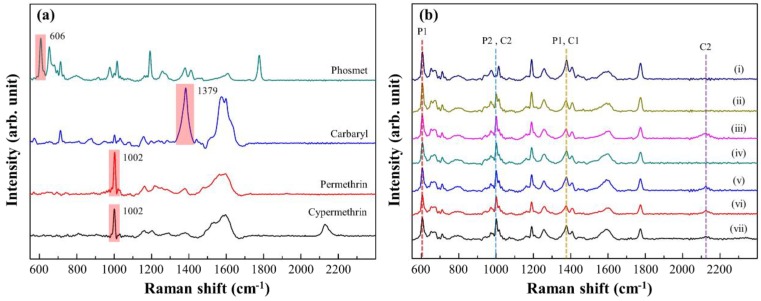
(**a**) The characteristic SERS peaks for the pesticides P1, C1, P2 and C2 at the concentration of 10^−3^ M; (**b**) SERS signals from 7 samples (i to vii) for the subsequent mixture of P1, C1, P2 and C2. Their characteristic peaks were identified.

Using Au NPs/ZrO_2_ NFs_0.3 as the substrate, in Figure 5a, the characteristic SERS peaks for P1, C1, P2 and C2 in 10^−3^ M were examined and taken as the references. The major characteristic peaks of P1 (606, 654, 713, 1192, 1379, 1407 and 1776 cm^−1^), C1 (713, 1379, 1441 and 1582 cm^−1^), P2 (1002, 1017, 1162, 1209 and 1582 cm^−1^) and C2 (1002, 1017, 1162, 1209, 1582 and 2130 cm^−1^) were identified. The most intense peaks of P1 (606 cm^−1^, δ(C=O)), C1 (1379 cm^−1^, symmetric ring vibration), P2 (1002 cm^−1^, benzene ring breathing vibration) and C2 (1002 cm^−1^, benzene ring breathing vibration) were respectively determined at low concentrations of pesticides. The limits of detection were estimated as 10^−6^ M for cypermethrin (C2), 10^−7^ M for carbaryl (C1) and permethrin (P2), and 10^−8^ M for phosmet (P1), which in practice, meet the trace concentration requirement of, for example, food security [23]. There are five samples per test group. Each sample is performed from 20 spots. The results of using these concentrations can be found in the Supporting Data 4–8.

The application of SERS to analyze multiple pesticides (in 10^−3^ M) was carried out using Au NPs/ZrO_2_ NFs_0.3 as the substrate. The characteristic peaks of each pesticide, shown in Figure 5a, were used as the references for the presence of the pesticides. In Figure 5b, qualitative measurements from seven identical solutions with multiple pesticides showed that Au NPs/ZrO_2_ NFs_0.3 was competent to distinguish the presence of four pesticides. Relatively low peak intensities naturally resulted from a low concentration of each pesticide. However, due to the overlapping of peaks, increases of relative peak intensities were found at 1002 cm^−1^ for P2 and C2, and at 1379 cm^−1^ for P1 and C1. 

ZrO_2_ shows a strong affinity to phosphoryl group [24], which could make organophosphates more competitive to adsorb on the ZrO_2_ surface. To verify the effect of ZrO_2_ templates on organophosphates, the reduction of Raman intensity for each pesticide in various mixtures compared to that in standard solutions is shown in Table 1. The reduction of Raman intensity of phosmet [25], which is an organophosphate, was less than the other pesticides in the mixtures. The results indicate that Au NPs/ZrO_2_ NFs_0.3 exhibited higher selectivity to organophosphates due to the property of ZrO_2_ templates. The utilization of ZrO_2_ NFs can be exploited as a substrate for enhancing Raman intensity on the adsorbed molecules. ZrO_2_ showed a strong affinity toward the phosphate group on parathion molecules, which provides sensitivity and selectivity of the sensing film [26], as nitroaromatic NPs strongly bind to the ZrO_2_ nanofiber surface [27]. The deposition of gold particles on the fibers further amplifies Raman signals due to SERS. This study suggests that Raman signals can be finely tuned in intensity and effectively enhanced in nanofiber mats and arrays by properly tailoring the architecture, composition and light-scattering properties of the complex networks of filaments. In addition, the large area of inter-Au NP surface plasmon resonance on ZrO_2_ NFs as a SERS-active substrate was applied to distinguish multiple pesticides due to their formation of hot spots (Figure 1).

### 3.4. Detection of Simulated Pesticides on Apples

**Figure 6:**
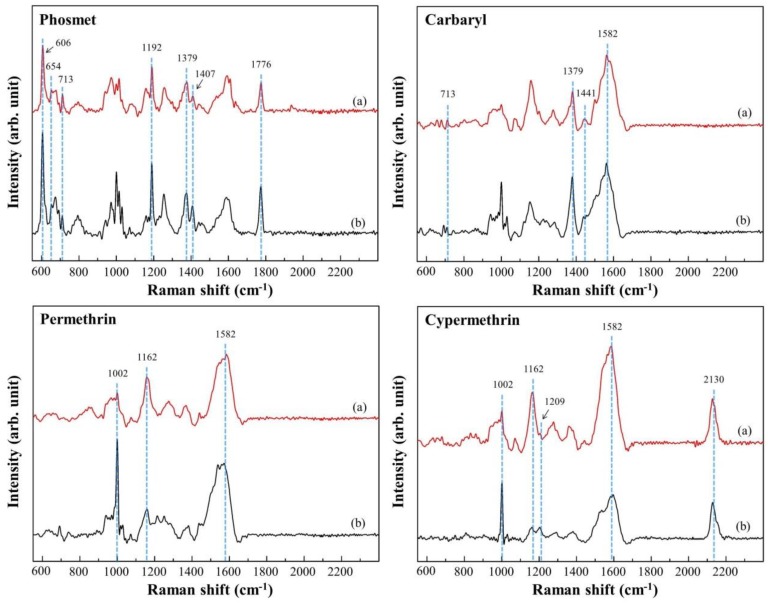
SERS spectra (a) from the pesticide-containing apple peels in comparison with (b) a standard solution.

According to world food ethics (which are based on USA food rules) [28], the agricultural crops contaminated with residual pesticides (C1, P1, C2, P2) are certainly harmful to human health [29]. To simulate the detection of pesticides on fruits, apples were spiked with 10^−2^ M standard solution containing C1, P1, C2 and P2. Subsequently, the apple peels were soaked in ethanol to extract pesticides. The concentration of each pesticide in extracts was calculated to 10^−4^ M. SERS spectra containing pesticides extracted from apple peels are shown in Figure 6a. The characteristic peaks in the obtained SERS spectra matched those in the SERS spectra from the standard solutions, as shown in Figure 6b. The enhancement was attributed to high EM effect, which was mainly induced by Au NPs. The SERS effect occurred at the metal–semiconductor junction and generated strong local electromagnetic (EM) field at the interface in the hybrid nanosystem [30]. Notably, the physical methods prevent samples from chemical or residual contaminations and provide a large density of Raman hot-spot areas. Our optimized Au NPs/ZrO_2_ NFs substrate was applied for pesticide detection.

## 4. Conclusions

To have a simple and fast detection of pesticide residue on fruits or vegetables, a novel material with a cost-effective solution has been proposed. Chemically stable ZrO_2_ NFs are prepared by a nonthermal spin-coated sol-gel method, followed by depositing Au NPs and forming as an integrated Au NPs/ZrO_2_ NFs substrate. The optimized sample Au NPs/ZrO_2_ NFs_0.3 has been proved to be SERS-active with an EF of 2.1 × 10^7^. Au NPs/ZrO_2_ NFs_0.3 is competent to distinguish the characteristic Raman peaks from four kinds of pesticide residue; their detection limits can be lowered to 10^−6^–10^−8^ M. Besides, Au NPs/ZrO_2_ NFs show high selectivity to organophosphates when multiple pesticides are present. For a practical application on the diluted juice containing sliced pesticide-containing apple peels, the characteristic peaks of each pesticide could also be clearly identified. Moreover, Au NPs/ZrO_2_ NFs can be made on a large surface area and are thus promising for flexible and extensible applications.

## Figures and Tables

**Table 1:** Raman intensity reduction of each pesticide in various mixtures compared to that in standard solutions.

Mixture ^a^	Raman Intensity Reduction (%) ^b^			
	P1	C1	P2	C2
i	04.0	68.1	-	-
ii	02.5	-	64.7	-
iii	18.9	-	-	39.4
iv	27.2	83.9	60.9	-
v	20.1	82.1	-	49.7
vi	33.6	-	94.3	60.2
vii	25.5	80.4	80.3	70.5

^a^ The concentration of each pesticide in various mixtures was 10^−3^ M; ^b^ the Raman intensity reduction was the ratio of Raman intensity of each pesticide in mixtures to that in standard solutions.

## Supplementary Materials

The following are available online at http://www.mdpi.com/2079-4991/8/6/402/s1, Figure S1: Supporting Data 1: (a) Low magnification and (b) high magnification of FE-SEM images for the sample AuNPs/ZrO_2_NFs_0.3. A uniform Au NPs are deposited onto ZrO_2_ NFs. Figure S2: Supporting Data 2: The EDS-mapping image of the cross-sectioned sample AuNPs/ZrO_2_NFs_0.3. The green spot is Au element, while the red spot is Zr one. Figure S3: Supporting Data 3: The size distribution histogram of Au NPs on the sample AuNPs/ZrO_2_NFs_0.3. To calculate the sizes, random 200 particles on the Supporting Data 1(a) are chosen. Figure 4S: Supporting Data 5: (a) SERS spectra of phosmet standard solutions of various concentrations, and (b) the molecular structure of phosmet. Figure 5S: Supporting Data 6 (a) SERS spectra of carbaryl standard solutions of various concentrations, and (b) the molecular structure of carbaryl. Figure 6S: Supporting Data 7: (a) SERS spectra of permethrin standard solutions of various concentrations, and (b) the molecular structure of permethrin. Figure 7S: Supporting Data 8 (a) SERS spectra of cypermethrin standard solutions of various concentrations, and (b) the molecular structure of cypermethrin. Table S1: Supporting Data 4: Raman spectra of the analytes.
