# Supplementary material for: Gold Nanoparticle-Coated ZrO2-Nanofiber Surface as a SERS-Active Substrate for Trace Detection of Pesticide Residue
Source: Nanomaterials (Basel). 2018 Jun 3;8(6):402. doi: 10.3390/nano8060402 (PMC6027227; doi:10.3390/nano8060402)
Supplement: Supplementary file 1 [file nanomaterials-08-00402-s001.pdf]

nanomaterials

MDPI

Article

Gold Nanoparticle-Coated ZrO_2-Nanofiber Surface as a SERS-Active Substrate for Trace Detection of Pesticide Residue

Han Lee [1], Jiunn-Der Liao [1,2,*], Kundan Sivashanmugan [1], Bernard Haochih Liu [1], Wei-en Fu [3], Chih-Chien Chen [1], Guo Dung Chen [3] and Yung-Der Juang [4]

1 Department of Materials Science and Engineering, National Cheng Kung University, 1 University Road, Tainan 701, Taiwan; rick594007@hotmail.com (H.L.); sivashanmugannst87@gmail.com (K.S.); hcliu@mail.ncku.edu.tw (B.H.L.); mvp820820@gmail.com (C.-C.C.)

2 Medical Device Innovation Center, National Cheng Kung University, 1 University Road, Tainan 701, Taiwan

3 Center for Measurement Standards, Industrial Technology Research Institute, No. 321, Kuang Fu Road, Sec. 2, Hsinchu 300, Taiwan; WeienFu@itri.org.tw (W.-e.F.); Eric_chen@itri.org.tw (G.D.C.)

4 Department of Materials Science, National University of Tainan, Tainan 700, Taiwan; juang@mail.nutn.edu.tw

* Correspondence: jdliao@mail.ncku.edu.tw; Tel.: +886-6-2757575 (ext. 62971); Fax: +886-6-2346290

Supporting Data 1

The SEM images after eposition are shown in Supporting Data 1 (a) with a magnification of 5×10^5. The results show that the Au NPs are *uniformly distributed* over the surfaces of ZrO_2 NFs. The Supporting Data 1 (b) shows a higher magnification SEM image with a magnification of 10^6, which may confirm our described results.

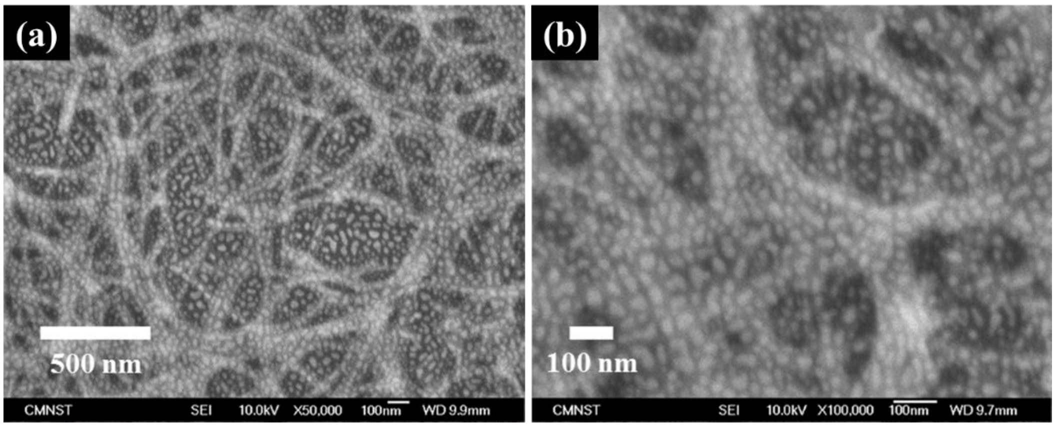

Supporting Data 1: (a) Low magnification and (b) high magnification of FE-SEM images for the sample AuNPs/ZrO_2NFs_0.3. A uniform Au NPs are deposited onto ZrO_2 NFs.

Supporting Data 2

In the Supporting Data 2, the EDS-mapping image of the cross-sectioned sample AuNPs/ZrO_2NFs_0.3 is shown. The green spot is Au element, while the red spot is Zr one.

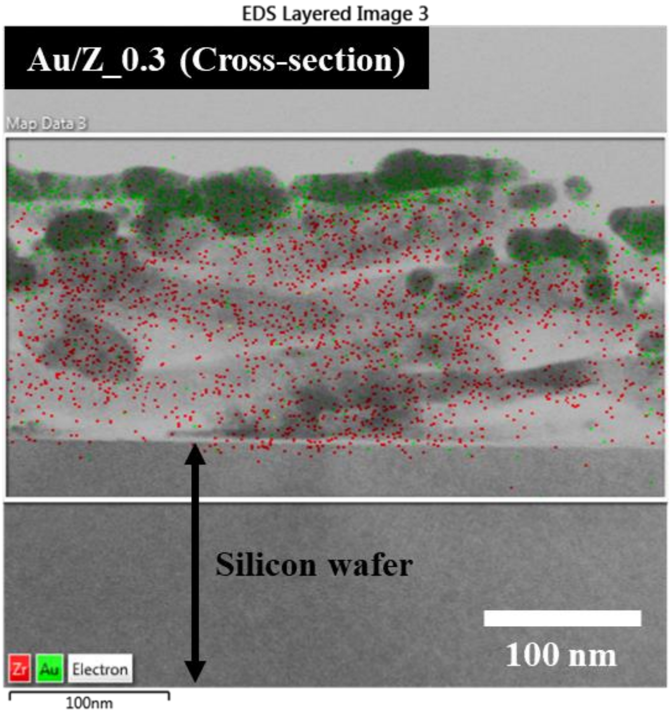

Supporting Data 2: The EDS-mapping image of the cross-sectioned sample AuNPs/ZrO_2NFs_0.3. The green spot is Au element, while the red spot is Zr one.

Supporting Data 3

The particle size distribution of the nano-Au NPs is also shown in the Supporting Data 3, in which the distribution ranges from 30 to 45 nm. A narrow distribution curve represents a uniform size of Au NPs.

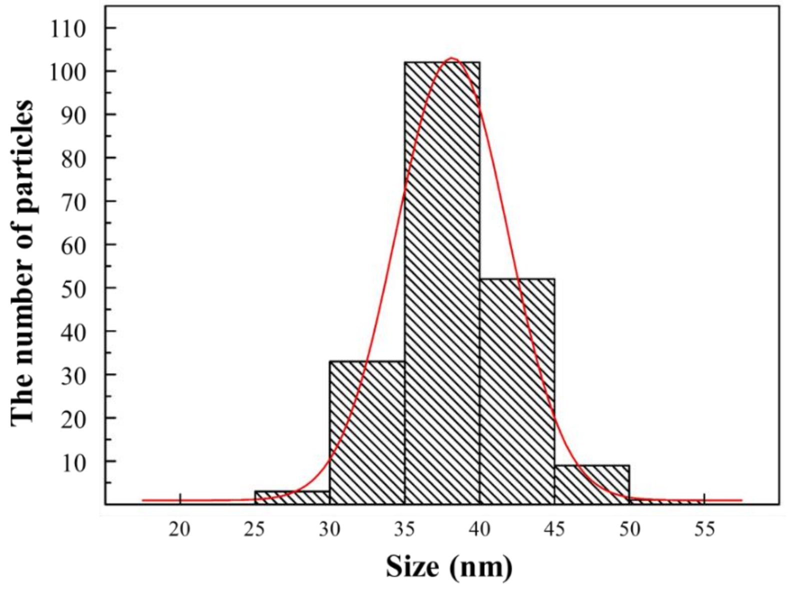

Supporting Data 3: The size distribution histogram of Au NPs on the sample AuNPs/ZrO_2NFs_0.3. To calculate the sizes, random 200 particles on the Supporting Data 1(a) are chosen.

Supporting Data 4

Table 1. Raman spectra of the analytes.

Pesticide	Raman shift (cm^{-1})	Assignment
Phosmet	501	ρ(CH_2) + ρ(PO_2), rocking vibration
	650	δ(C=O), in-plane deformation vibration
	712	benzene ring breathing
	1014	asymmetric stretching of P–O–C deformation vibration
	1189	δ (C–N), in-plane deformation vibration
	1381	δ(CH_3), in-plane deformation vibration
	1772	υ(C=O), stretching
Carbaryl	534	C–C bending
	713	δ(NCOC), in-plane deformation vibration
	1374	symmetric ring vibration
	1441	ω(C–H), non-planar rocking
	1582	υ(C=C), stretching in naphthalene ring
Permethrin	1002	benzene ring breathing
	1017	υ(C=O), stretching
	1162	υ(C=O), stretching
	1209	υ(C=O), stretching
	1582	υ(C=C), stretching
Cypermethrin	1002	benzene ring breathing
	1017	υ(C=O), stretching
	1162	υ(C=O), stretching
	1209	υ(C=O), stretching
	1582	υ(C=C), stretching
	2130	υ(C≡N), stretching

Supporting Data 5

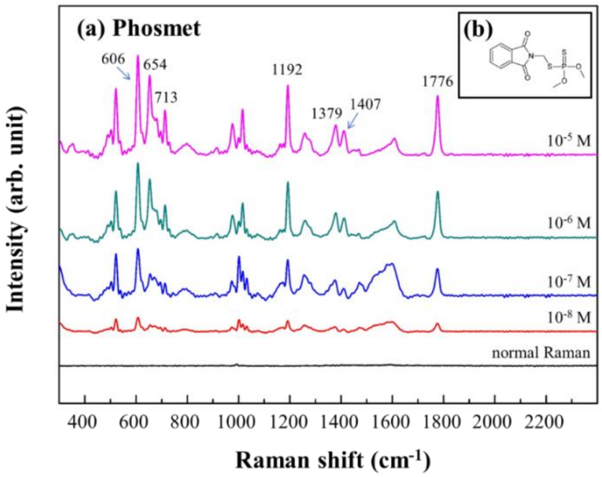

Supporting Data 5 (a) SERS spectra of phosmet standard solutions of various concentrations, and (b) the molecular structure of phosmet. The Raman characteristic peaks of phosmet, including 606 (δ(C=O)), 654 (δ(P=S)), 713 (breath of benzene ring), 1192 (δ(C–N)), 1379 (δ(CH_3)), 1407 (γ(C–H) in S–CH_2–N), and 1776 cm^{-1} (υ(C=O)) could be observed.

Supporting Data 6

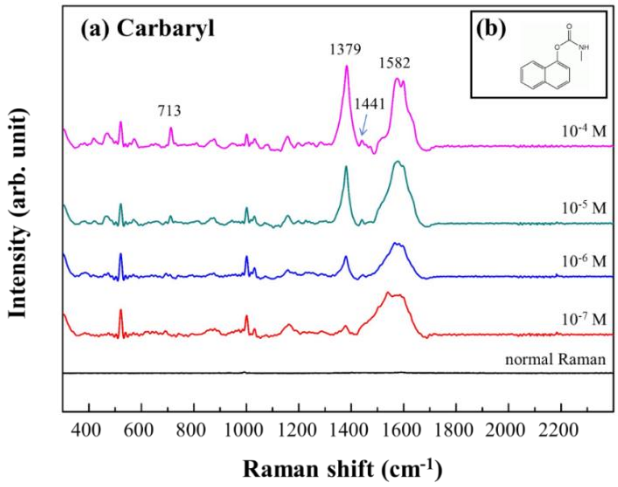

Supporting Data 6 (a) SERS spectra of carbaryl standard solutions of various concentrations, and (b) the molecular structure of carbaryl. The Raman characteristic peaks of carbaryl, including 713 (δ(NCOC)), 1379 (symmetric ring vibration), 1441 (ω(C–H)), and 1582 cm^{-1} (υ(C=C) in naphthalene ring) could be observed.

Supporting Data 7

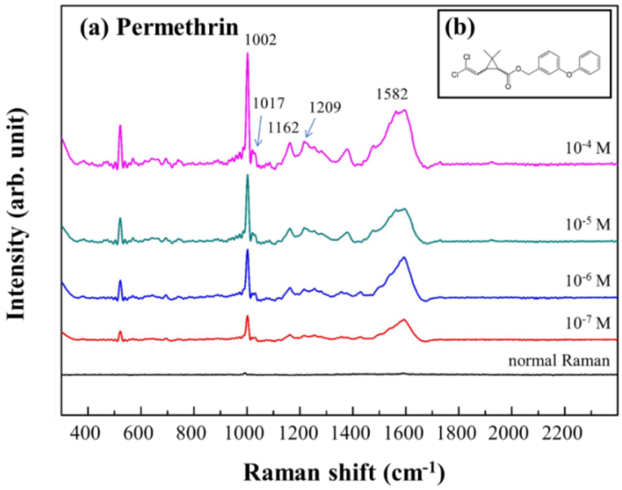

Supporting Data 7 (a) SERS spectra of permethrin standard solutions of various concentrations, and (b) the molecular structure of permethrin. The Raman characteristic peaks of permethrin, including 1002 (breath of benzene ring), 1017 (υ(C–O)), 1162 (υ(C–O)), 1209 (υ(C–O)), and 1582 cm^{-1} (υ(C=C)$_{ben.}$) could be observed.

Supporting Data 8

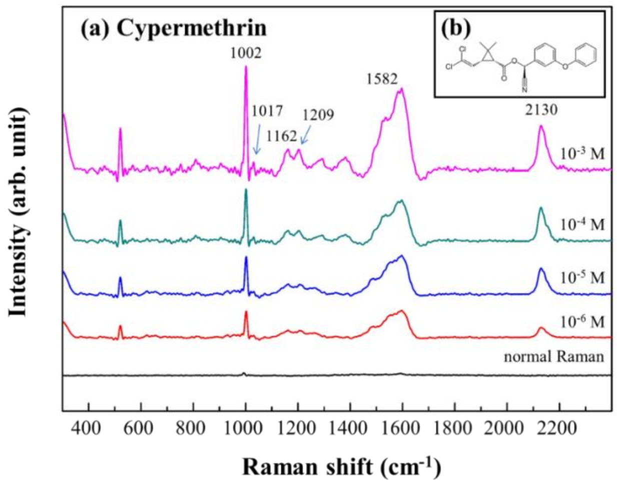

Supporting Data 8 (a) SERS spectra of cypermethrin standard solutions of various concentrations, and (b) the molecular structure of cypermethrin. The Raman characteristic peaks of cypermethrin, including 1002 (breath of benzene ring), 1017 (υ(C–O)), 1162 (υ(C-O)), 1209 (υ(C–O)), 1582 ($\upsilon(C{=}C)_{ben.}$), and 2130 cm^{-1} (υ(C≡N)) could be observed.
